# Consumption of fruits and vegetables and risk of renal cell carcinoma: a meta-analysis of observational studies

**DOI:** 10.18632/oncotarget.15841

**Published:** 2017-03-02

**Authors:** Shaojing Zhang, Zhankui Jia, Zechen Yan, Jinjian Yang

**Affiliations:** ^1^ Department of Urology Surgery, The First Affiliated Hospital, Zhengzhou University, Zhengzhou, Henan Province, China

**Keywords:** fruits and vegetables, renal cell carcinoma, meta-analysis, relative risk

## Abstract

**Background:**

There have been inconsistent results about the association between consumption of fruits and vegetables and renal cell carcinoma (RCC) risk. We conducted a meta-analysis of the published observational studies to explore this association.

**Results:**

Nineteen observational studies (4 cohort, 1 pooled and 14 case-control studies), involving 10,215 subjects with RCC were part of this meta-analysis. The SRR for the highest vs. the lowest intake of vegetables was 0.73 (95% CI: 0.63–0.85; P_heterogeneity_ = 0.004, I^2^ = 53.5%), whereas for fruits it was 0.86 (95% CI: 0.75–0.98; P_heterogeneity_ = 0.012, I^2^ = 47.4%). Linear dose-response analysis also showed similar results, e.g., for per 1 serving/day increment of vegetables, the SRR was 0.90 (95% CI: 0.84–0.96) and for fruits it was 0.97 (95% CI: 0.93–1.01). Nonlinear association was only observed for vegetables (P_nonlinearity_ = 0.001), but not for fruits (P_nonlinearity_ = 0.221).

**Materials and Methods:**

Eligible studies up to August 31, 2016 were identified and retrieved by searching MEDLINE and EMBASE databases along with manual review of the reference list from the retrieved studies. Quality of included studies was evaluated using Newcastle-Ottawa Quality Assessment Scale (NOS). Random-effects model was used to calculate summary relative risk (SRR) and corresponding 95% confidence interval (CI).

**Conclusions:**

This meta-analysis indicated a protective effect of consumption of vegetables and fruits on RCC risk. Further studies are warranted with prospective designs that use validated questionnaires and control for important confounders.

## INTRODUCTION

In the United States, kidney cancer is estimated as the tenth-and seventh-highest incident cancers among women and men, respectively [1], with annual increments of 1.7 and 1.6 % in white women and white men. Renal cell carcinoma (RCC) is the most common malignancy of the kidney [2]. Globally, geographic variation in RCC demonstrates higher age standardized incidence rates in more developed areas (11.9/10^5^). compared to developing regions (2.5/10^5^) [3] Although smoking [4], obesity [5], hypertension [6], diabetes [7] and some medications [8] have consistently been associated with RCC risk, the exact etiology remains largely unknown.

Consumption of vegetables and fruits (VFs), which contains putative anticarcinogenic and antimutagenic substances (e.g., vitamin C, vitamin E, folate, carotenoids and flavonoids), have long been thought to protect against cancers, including RCC. The suggested mechanisms for prevention of cancer includes: induce detoxifying phase II enzymes, antioxidant activity, protection against DNA damage, modulate DNA methylation, promotion of apoptosis [9]. A large number of epidemiological studies reporting on associations between consumption of VFs and RCC risk have given inconclusive results [10–31]. According to the 2015 WCRF/AICR Continuous Update Project Report, no conclusions can be reached for the evidence of VFs consumption and RCC incidence [32]. Results from several large prospective cohort [11, 12, 19, 31] and case-control studies [21, 23, 25–27, 30] have shown no associations between VFs intake and RCC risk. In contrast, data from a pooled analysis of 13 cohort studies [15] observed that high fruit and vegetable consumption was associated with a decreased risk of RCC. Since this pool analysis was published, additional two large cohort studies [11, 12] have been available. In the current analysis, we also included case-control studies to increase statistical power, and we examined the exact shape of the dose-response relationship between consumption of VFs and RCC risk. We performed study quality assessment in detail and meta-regression and sensitivity analyses according to the study variables.

## RESULTS

### Search results and study characteristics

**Figure 1:**
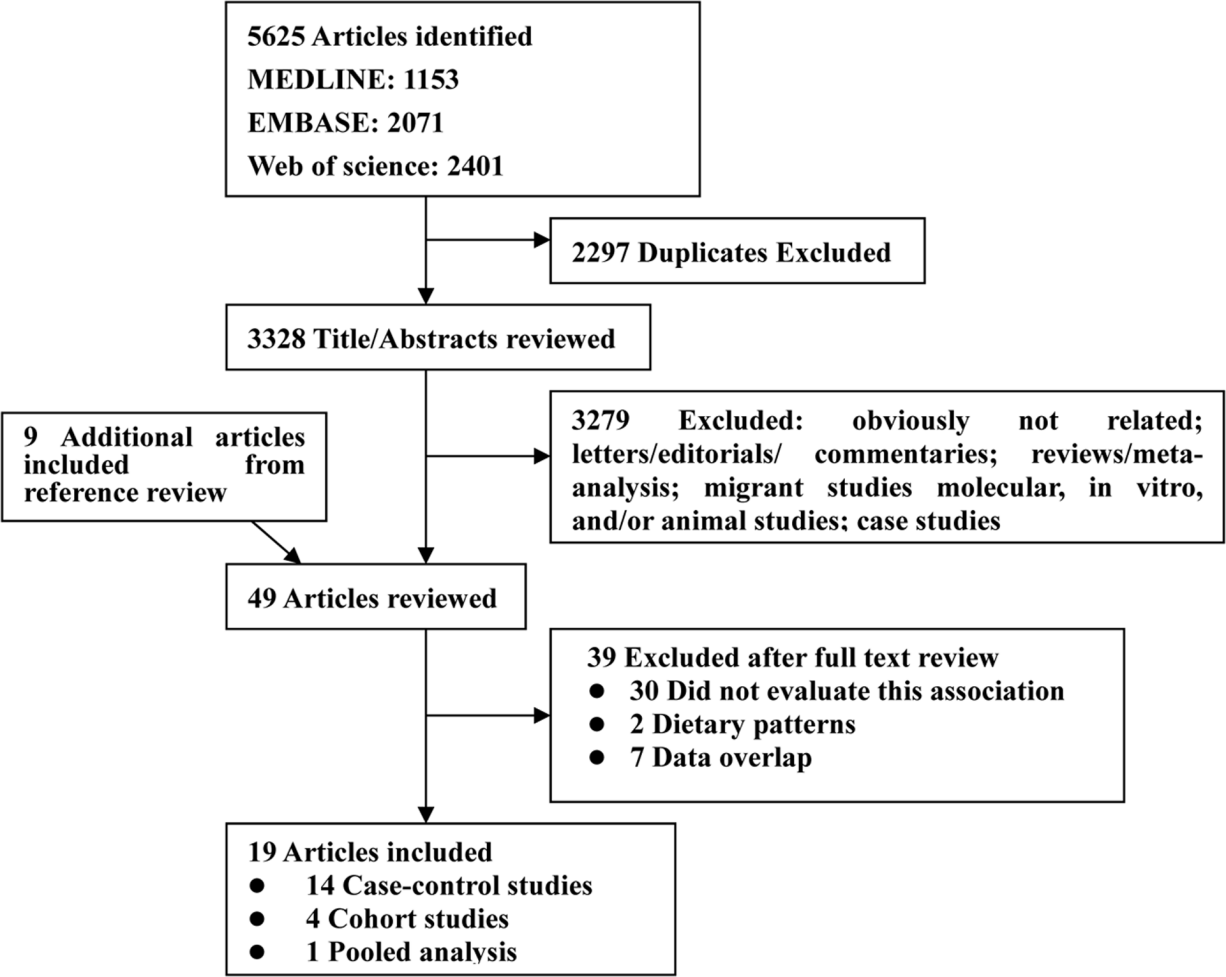
Flow diagram of systematic literature search on vegetables and fruit intake and the risk of renal cell carcinoma

Based on the study selection criteria, we identified 1153 articles from the MEDLINE, 2071 from the EMBASE and 2401 from the web of science database. In addition, 9 more articles were identified by studying the cross-reference lists. After excluding studies that did not meet the inclusion criteria, 19 publications were included in our meta-analysis, including 14 case-control studies, a pooled analysis of 13 prospective studies and 4 cohort studies (Figure 1). We excluded five publications [14, 20, 33–35] in the primary analysis (because they were included in the aforementioned pooled analysis [15]), but included them in the subgroup analysis for genders.

Table 1 and Table 2 depicted the characteristics of these 19 publications. A total of 10 215 cases and 1 394 677 controls/participants were enrolled in these studies. All these studies represented different populations: 7 studies were conducted in North America (United States and Canada), 8 in Europe, one each in Asia (China) and in South America, respectively. Two studies were multinational (Europe and USA), and 1 was pooled study. Only 4 studies [11, 12, 15, 17] adjusted for all the three main risk factors for RCC (16 studies adjusted for tobacco smoking, 15 for BMI and 4 for hypertension). The quality scores of each study are summarized in Supplementary Table 1. The quality scores ranged from 5 to 9, with the median score of 8. The majority of included studies (16/19) were of high quality (NOS score ≥ 7).

**Table 1:** Characteristics of case-control studies of vegetables and fruit intake and renal cell carcinoma risk

Author/ year, Country	Number of cases and controls Age, mean or range, sex	Case ascertainment	Dietary assessments	Exposure details	RR (95% CI) (Highest vs. lowest)	NOS	Adjustments
**Population based**							
Brock et al., 2012, USA [13]	RCC, *N =* 323 Age: 40–85 yr; M (63.0%) Controls, *N =* 1827	Histological	Self-administered Validated FFQ-55	V: > 2.1 vs. 0–1 servings/d F: > 2.4 vs. 0–1 servings/d	0.5 (0.3–0.7) 0.7 (0.4–1.1)	9	Age, sex, proxy status, years of smoking, blood pressure, alcohol consumption, fat consumption and energy
Grieb et al., 2009, USA [16]	RCC, *N =* 335 Age: 66 yr; M (54.0%) Controls, *N =* 337	Histological	Interview FFQ-70 Validated	V: Q4 vs. Q1 F: Q4 vs. Q1	0.49 (0.25–0.96) 0.73 (0.46–1.17)	8	Age, sex, race, income, BMI, smoking
Handa et al., 2002, Canada [21]	RCC, *N =* 461 Age:35–74 yr; M (54.4%) Controls, *N =* 672	Histological	Self-administered FFQ-69 NA	F:Q4 vs. Q1	1.3 (0.8–2.1)M	7	Age, smoking status, BMI
Lindblad et al., 1997, Sweden [23]	RCC, *N =* 542 Age: 64.1 yr; M (63.0%) Controls, *N =* 493	Histological	Interview FFQ-63 NA	V: > 816 vs. < 219 g/wk F: > 1907 vs. 576 g/wk	0.84 (0.53–1.31) 0.65 (0.42–1.02)	7	Age, sex, BMI, cigarette smoking, and educational level.
Boeing et al., 1997, German [24]	RCC, *N =* 277 Age: 20–75 yr; M (64.6%) Controls, *N =* 286	Histological	Self-administered FFQ-122 NA	V: T3 vs. T1 F: T3 vs. T1	0.75 (0.44–1.27) 0.40 (0.23–0.69)	6	Age, sex, educational status, tobacco smoking and alcohol consumption
Wolk et al., 1996, multi centers [25]	RCC, *N =* 1185 Age: 62.5 yr; M (63.0%) Controls, *N =* 1526	Histological	Self-administered and interview FFQ, NA	V: Q4 vs. Q1 F: Q4 vs. Q1	0.81 (0.61–1.08) 0.85 (0.66–1.10)	7	Age, sex, stud center, BMI, smoking, total calories
Mellemgaard et al., 1996, Denmark [26]	RCC, *N =* 351 Age: 20–79 yr; M (61.5%) Controls, *N =* 340	Histological	Self-administered FFQ-92 validated	V: ≥ 1 vs. < 0 servings/wk F: > 3 VS. < 1 times/wk	0.6 (0.2–1.7)M 1.0 (0.4–2.5)W 0.6 (0.3–1.4) M 0.9 (0.4–2.3)W	9	Age, smoking, BMI, and socio-economic status.
Chow et al., 1994, USA [27]	RCC, *N =* 690 Age and sex: NA Controls, *N =* 707	Histological	Self-administered FFQ-65 validated	V: Q4 vs. Q1 F: Q4 vs. Q1	1.0 (0.7–1.5) 1.2 (0.8–1.7)	8	Age, sex, cigarette smoking, and BMI.
McLaughlin et al., 1992, China [28]	RCC, *N =* 154 Age: 60.5 yr; M (58.4%) Controls, *N =* 157	Histological	Interview questionnaire NA	V: Q4 vs. Q1 F: Q4 vs. Q1	0.3 (0.1–0.7)M 1.5 (0.6–4.6)W 0.2 ( 0.0–0.5)M 0.7 ( 0.2–2.0)W	9	Age, education, cigarette smoking, BMI
**Hospital based**							
Bravi et al., 2007, Italy [18]	RCC, *N =* 767 Age: 62 yr; M (64.4%) Controls, *N =* 1534	Histological	Interview FFQ-78 validated	V: > 15.7 vs. 6.2 servings/wk F > 18.4 vs. < 6.4 servings/wk	0.65 (0.47–0.90) 1.02 (0.76–1.37)	7	Age, center, sex, period of interview, education, smoking, alcohol drinking, BMI, family history of kidney cancer, total energy intake
Hsu et al., 2007, Eastern and Central Europe [17]	RCC, *N =* 1065 Age: 20–79 yr; M (58.4%) Controls, *N =* 1509	Histological	Interview FFQ-23 validated	V: > 1 times/wk vs. < 1 times/month	0.64 (0.51–0.80)	7	Age, country, sex, smoking, education, BMI, hypertension medication use, alcohol consumption, total red meat and total white meat consumption
De Stefani et al., 1998, Uruguay [22]	RCC, *N =* 121 Age: 30–89 yr; M (60.3%) Controls, *N =* 243	Histological	Interview FFQ-23 validated	V: > 157 VS. < 52 servings/yr F: > 313 vs. < 104 servings/yr	0.46 (0.24–0.88) 1.66 (0.93–2.96)	7	Age, sex, residence, urban-rural status, education, BMI, mate drinking.
Negri et al., 1991, Italy [29]	RCC, *N =* 147 Age: 20–74 yr; M (66.0%) Controls, *N =* 6147	Histological	Interview FFQ NA	V: ≥ 7 vs. < 7servings/wk F: ≥ 14 vs. < 7 servings/wk	0.4 (0.2–0.7) 0.6 (0.4–1.0)	7	Age, residence, education, smoking
Talamini et al., 1990, Italy [30]	RCC, *N =* 240 Age: 58.0 yr; M (62.5%) Controls, *N =* 665	Histological	Interview FFQ-14 NA	V: T3 vs. T1 F: T3 vs. T1	1.18 (0.73–1.91) 0.92 (0.63–1.35)	5	Age, sex, education, area of residence, BMI
Abbreviation: BMI, body mass index; FFQ, food frequency questionnaire; NA, not available, V, vegetable; F, fruit; M, men, W, women; RCC, renal cell carcinoma; T, tertile; Q4, quartitle; M, male.							

**Table 2:** Characteristics of prospective cohort studies of vegetables and fruit intake and renal cell carcinoma risk

Author/ year, Country	Study name, number of subjects, FU	Dietary assessments	Exposure details	Case ascertainment Cases (*n*)	RR (95% CI) (Highest vs. lowest)	NOS	Adjustments
Macleod et al., 2013, USA [11]	VITAL Cohort *N =* 77260 Age: 50–76 yr; M (64.4%) FU, 8 yr	Self-administered, validated FFQ	V: T3 vs. T1 F: T3 vs. T1	Cancer registry 249 RCC	0.76 (0.52–1.11) 1.02 (0.71–1.46)	9	Age, sex, alcohol, obesity, smoking, hypertension, history of kidney disease, viral hepatitis, DM,
Daniel et al., 2013, USA [12]	NIH-AARP Diet and Health Study *N =* 491,841 Age: 62 yr; M (59.6%) FU, 9 yr	Self-administered Validated FFQ-124	V: 1.83 vs. 0.52 servings/1000k/d F:2.26 vs. 0.3 servings/1000k/d	Cancer registry 1816 RCC	0.97 (0.84–1.12) 0.98 (0.84–1.15)	9	Age, sex, education, race, marital status, family history of any cancer, BMI, smoking status, hypertension, diabetes, alcohol, red meat, and total energy, legumes, whole grains
Lee et al., 2009, Europe and USA [15]	13 cohorts *N =* 774,952 Age: 22–104 yr; M (52.0%) FU, 7–20 yr	Self-administered Validated FFQ	V: Q5 vs. Q1 F: Q5 vs. Q1	Medical records, cancer registries 1478 RCC	0.80 (0.67–0.95) 0.87 (0.69–1.08)	9	Age, hypertension, BMI, smoking, combination of parity and age at first birth, alcohol intake, and total energy intake
Weikert et al., 2006, Europe [19]	EPIC *N =* 375851 Age: 62 yr; M (60.3%) FU, 6.2 yr	Self-administered Validated FFQ	V: per 40/g/d F: per 40/g/d	Cancer or mortality registries 306 RCC	0.97 (0.85–1.11) 1.03 (0.97–1.08)	9	Age, center, BMI, energy from fat sources, energy from non-fat sources, education, smoking, alcohol drinking
Fraser et al., 1990, USA [31]	California Seventh-day Adventists *N =* 34198 Age: 72.3 yr; M (60.3%) FU, 6.2 yr	Self-administered Validated FFQ-51	F: > 3 vs. < 3 servings/wk	Mortality registries 14 RCC	0.21 (0.05–1.45)	6	Age, sex

Abbreviation: VITAL, the VITamin And Lifestyle; DM, diabetes mellitus, BMI, body mass index; FFQ, food frequency questionnaire; NA, not available, V, vegetable; F, fruit; M, men, W, women; RCC, renal cell carcinoma; FU, follow-up; T, tertile; Q5, quintitle.

### Total vegetables

#### High vs. low analysis

**Figure 2:**
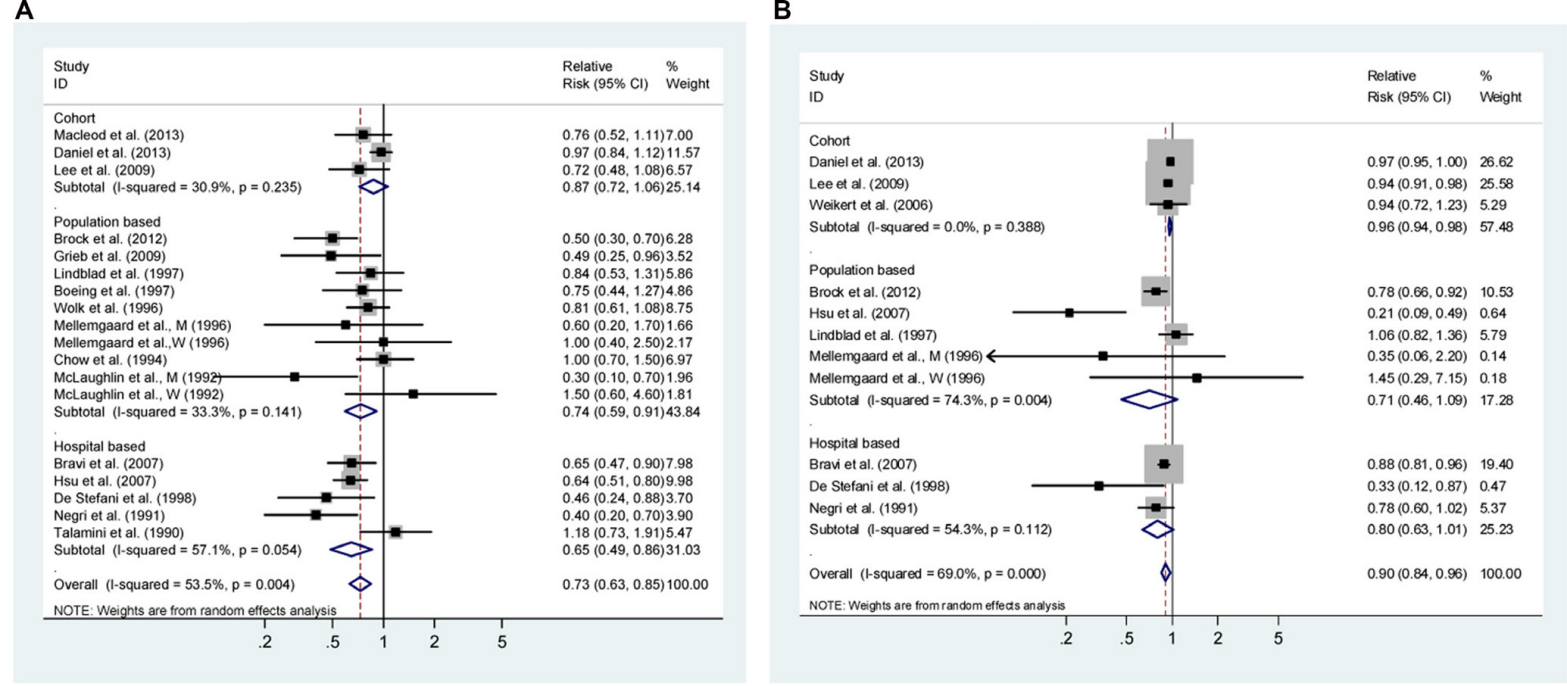
Analysis of vegetables intake with risk of renal cell carcinoma (**A**) High *vs*. Low analysis; (**B**) Dose-response analyses for intake in increment of 1 serving/day. Studies are sub-grouped according to study design. Squares indicated study-specific risk estimates (size of square reflects the study-statistical weight, i.e. inverse of variance); horizontal lines indicate 95% confidence intervals; diamond indicates summary relative risk estimate with its corresponding 95% confidence interval.

Sixteen studies investigated the association between the highest *vs*. lowest vegetables intake and RCC risk. The observed SRR was 0.73 (95% CI: 0.63–0.85), with moderate heterogeneity (P_heterogeneity_ = 0.004, I^2^ = 53.5%; Figure 2A).

#### Dose-response analysis

Dose-response analysis was done based on the data from ten studies (Figure 2B). The SRR per 1 serving/day was 0.90 (95% CI: 0.84–0.96), with evidence of moderate heterogeneity (I^2^ = 69.0%, P_heterogeneity_ < 0.001). There was evidence of a non-linear association between vegetable intake and RCC risk (*P* = 0.001 for non-linearity, Supplementary Figure 1A), with a significant reduction in RCC risk when increasing the intake up to about 3 servings/d intake of vegetables. Higher intake was associated with a further, but more modest decrease in risk.

### Total fruit

#### High vs. low analysis

**Figure 3:**
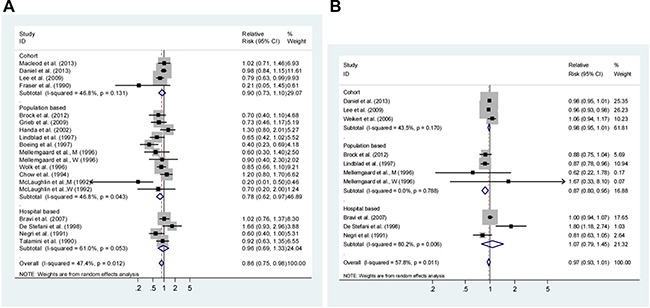
Analysis of fruits intake with renal cell carcinoma risk (**A**) High *vs*. Low intake and; (**B**) Dose-response analyses for intake in increment of 1 serving/day. Studies are sub-grouped according to study design. Squares indicated study-specific risk estimates (size of square reflects the study-statistical weight, i.e. inverse of variance); horizontal lines indicate 95% confidence intervals; diamond indicates summary relative risk estimate with its corresponding 95% confidence interval.

Eighteen studies representing the association between the highest *vs*. lowest fruits intake and RCC risk were used for this analysis. The SRR was 0.86 (95% CI: 0.75–0.98), and had low heterogeneity (P_heterogeneity_ = 0.012, I^2^ = 47.40%; Figure 3A).

#### Dose-response analysis

Dose-response analysis was achieved by including ten studies (Figure 3B). The SRR per 1 serving/day increment was 0.97 (95% CI: 0.93–1.01), with moderate heterogeneity (I^2^ = 57.8%, P_heterogeneity_ = 0.011). There was a linear association between fruits intake and RCC risk (*P* = 0.221 for non-linearity, Supplementary Figure 1B).

#### Subgroup, meta-regression and sensitivity analyses

Subgroup and meta-regression analyses were shown in Table 3. For high *vs*. low intake of vegetables, overall, there were inverse risk associations for RCC in all strata, but the associations were not statistically significant in cohort studies (SRR 0.87, 95% CI: 0.72–1.06), low study quality score (SRR 0.95, 95% CI: 0.61–1.49) and in studies using non-validated FFQ (SRR 0.76, 95% CI: 0.56–1.03). Adjustments for BMI significantly attenuated the protective role of vegetables consumption (*P* = 0.081). Locations, study design, study quality and confounders adjusted for smoking, alcohol use, history of hypertension and energy intake did not significantly alter the summary risk estimates (Table 3).

**Table 3:** Subgroup analyses of vegetables and fruit intake and renal cell carcinoma risk, high vs. low

Sub-groups	Vegetables					Fruit				
	Studies,*n*	SRR (95% CI)	*P*_h_	I^2^(%)	*P*_d_	Studies, *n*	SRR (95% CI)	*P*_h_	I^2^(%)	*P*_d_
All	16	0.73 (0.63–0.85)	0.04	53.5		18	0.86 (0.75–0.98)	0.012	47.4	
**Design**					0.252					0.763
Cohort	3	0.87 (0.72–1.06)	0.235	30.9		4	0.90 (0.73–1.10)	0.131	46.8	
Case-control	13	0.70 (0.59–0.82)	0.042	42.4		14	0.84 (0.70–0.99)	0.014	50.1	
**Sources of control**					0.527					0.329
Population-based	8	0.74 (0.59–0.91)	0.141	33.3		10	0.78 (0.63–0.97)	0.043	46.8	
Hospital-based	5	0.65 (0.49–0.86)	0.054	57.1		4	0.96 (0.69–1.33)	0.053	61.0	
**Geographic locations**					0.839					0.118
Europe	7	0.71 (0.59–0.87)	0.203	28.2		7	0.72 (0.56–0.93)	0.061	50.1	
North America	6	0.76 (0.57–1.03)	0.014	68.0		7	0.97 (0.81–1.16)	0.171	33.7	
South America	1	0.46 (0.24–0.88)	-	-		1	1.66 (0.93–2.96)	-	-	
Asia (China)	1	0.67 (0.14–3.22)	-	-		1	0.49 (0.16–1.48)	-	-	
**Gender**					0.158					0.957
Men	5	0.59 (0.32–1.03)	0.004	73.9		6	0.74 (0.51–1.07)	0.100	45.9	
Women	6	0.98 (0.71–1.35)	0.290	19.0		6	0.79 (0.59–1.06)	0.925	0	
**Type of FFQ**					0.647					0.194
Validated	10	0.71 (0.60–0.85)	0.008	58.1		11	0.93 (0.81–1.07)	0.127	34.0	
Not available	6	0.76 (0.56–1.03)	0.049	52.7		7	0.74 (0.57–0.96)	0.032	54.4	
**Data available**					0.199					0.847
Self-administered	7	0.82 (0.70–0.97)	0.146	35.4		10	0.87 (0.72–1.06)	0.022	53.8	
Interview	8	0.65 (0.52–0.83)	0.040	50.6		7	0.84 (0.65–1.07)	0.072	46.2	
**Study quality score**					0.250					0.149
High (NOS score > 6)	14	0.71 (0.60–0.83)	0.004	55.5		15	0.90 (0.79–1.01)	0.084	34.8	
Low (NOS score ≤ 6)	2	0.95 (0.61–1.49)	0.215	35.1		3	0.53 (0.25–1.14)	0.020	74.6	
**Adjustments**										
BMI, yes	14	0.77 (0.67–0.90)	0.020	48.0	**0.081**		0.93 (0.83–1.04)	0.182	24.6	**0.007**
No	2	0.54 (0.39–0.75)	0.289	19.5			0.54 (0.39–0.75)	0.313	15.7	
Smoking, yes	14	0.73 (0.62–0.84)	0.008	51.9	0.686	15	0.84 (0.73–0.96)	0.030	44.1	0.376
no	2	0.76 (0.30–1.90)	0.022	80.8		3	0.96 (0.47–1.94)	0.041	68.6	
Dietary energy, yes	5	0.74 (0.59–0.94)	0.013	68.3	0.832	5	0.90 (0.81–1.01)	**0.376**	5.5	0.748
No	11	0.72 (0.59–0.88)	0.055	42.0		13	0.82 (0.65–1.03)	0.005	56.2	
Hypertension, yes	5	0.72 (0.56–0.93)	0.004	74.4	0.957	4	0.90 (0.78–1.04)	0.287	20.6	0.773
No	11	0.73 (0.60–0.89)	0.064	40.5		14	0.83 (0.68–1.01)	0.001	53.5	
Alcohol use, yes	7	0.72 (0.59–0.87)	0.008	65.4	0.795	6	0.84 ( (0.69–1.03)	0.025	61.2	0.855
No	9	0.74 (0.58–0.94)	0.038	47.9		12	0.86 (0.71–1.05)	0.046	43.6	

For high *vs*. low intake of fruits, there were not statistically inverse associations for RCC risk in most of the strata. The association of high vs. low intake of fruits with RCC risk suggested inverse associations in the studies conducted in European countries (SRR, 0.72, 95% CI: 0.56–0.93), but not in North America (SRR, 0.97, 95% CI: 0.81–1.16). The SRR (95% CIs) estimates were 0.90 (0.73–1.10) for cohort studies, 0.78 (0.63–0.97) for population-based and 0.96 (0.69–1.33) for hospital-based case-control studies. Adjustments for BMI (*P* = 0.007) significantly attenuated the protective role of fruits consumption. Whereas geographic locations, study design, study quality, type of FFQ, methods of exposure available and adjustments for confounders (energy intake, alcohol use, hypertension and smoking) did not significantly modify the summary risk estimates for fruit intake (Table 3).

Meta-regression analyses showed that confounders adjusted by BMI were significant factors for the associations, which might account for 33.5% of the total between-study heterogeneity for vegetables intake and 60.7% for fruits intake. If the overall homogeneity and effect size were calculated by removing one study at a time, we confirmed the stability of the inverse association between consumption of vegetable and fruit and RCC risk (Supplementary Figure 2A–2B).

#### Publication bias

**Figure 4:**
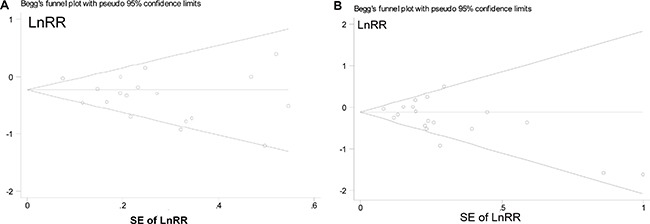
Begg's funnel plots of the log relative risks (RRs) versus the SEs of the log RRs in studies that evaluated the effect of vegetables (**A**) and fruit (**B**) intakes on the risk of renal cell carcinoma.

For high *vs*. low analysis, there was no evidence of publication bias for the risk association of RCC development with intake of vegetables (P_Begg's test_ = 0.705, Figure 4A; and P_Egger's test_ =0.667) and fruit (P_Begg's test_ = 0.142, Figure 4B; and P_Egger's test_ =0.633).

## DISCUSSION

Results of this meta-analysis indicate the hypothesis that consumption of vegetables and fruits may lower the risk of RCC development. The significant associations for vegetables and fruits were observed in case-control, but not in cohort studies. The association did not seem to differ among groups defined by geographical area, methods of dietary assessment and adjustments by smoking, history of hypertension, and total energy intake, but was significantly modified by adjustment by BMI/obesity. To our knowledge this is the first report suggesting a non-linear inverse association between consumption of vegetables and RCC risk, but the risk reductions leveled off for daily intake > 3 servings.

It has been proposed that several components rich in fruit and vegetables may lower the risk of RCC development. Carotenoids (α-carotene, β-carotene, lycopene and lutein/zeaxanthin) might inhibit oxidative damage to DNA, mutagenesis, tumor growth, malignant transformation [36]. Lycopene intake was inversely associated with RCC risk in prospective cohort studies (the highest vs. the lowest quartile of intake, HR = 0.61; 95% CI, 0.39–0.97) [37], although no association was observed by other studies [38, 39]. Dietary fiber is rich in fruit and vegetables. Some studies [12, 40], although not all [23, 25, 41], have found that high intake of fiber may lower the risk of RCC development.

Associations between intake of vegetables and fruits and RCC risk have been inconsistently reported among different observational studies. The results from most of the case-control studies showed non-significant inverse associations. Two multicenter case-control studies (each included more than 1,000 RCC cases) have shown more consistent inverse associations for consumption of fruits and vegetables. The Pooling Project analysis included data from approximately 1,500 RCC cases across 13 international cohorts, and showed that increasing fruit and vegetable consumption is associated with decreasing risk of RCC (*P* value, test for trend = 0.03 and 0.07, respectively) [15]. In contrast, other prospective studies [11, 12, 19] observed non-significant associations. When summarized risk estimation stratified by study design, we found a statistically significant association for consumption of fruits and vegetables among case-control studies, but a non-significant inverse association among prospective cohort studies. We assumed that this was due to several factors, e.g., potential selection and recall biases derived from a case-control design [42], the limitations of currently available dietary assessment tools, and the potential confounders for which analyses were adjusted. In addition, we found a non-significant association between intake of fruits and RCC risk among hospital-based case-control studies. This result should be treated with caution because this design is more subject to selection bias than the population-based design, and because there were only four hospital-based case-control studies for this association, which is subject to low statistical power. Likely, non-significant associations were also observed for both men and women when we combining results specifically for genders. These null associations may be due to low statistical power due to the small number of studies included.

In the present analysis, we captured the maximal number of published studies on this topic by using multiple avenues for finding articles, including several prospective studies. Additionally, a large sample size (10,215 cases and 1,394,677 controls/ participants) were recruited in our studies, and thus have a much greater possibility of reaching detecting smaller associations.

However, there are several limitations of this meta-analysis. Misclassification of VF intake may have an influence on our findings. It is very difficult for persons to accurately report their intake levels of VF. Compared with food records or food diaries, using a FFQ showed spearman's correlation coefficients of 0.6–0.8 for fruits consumption and only 0.19–0.62 for vegetables consumption [15, 42–44]. In addition, FFQ was validated in most of the included studies, and subgroup analysis suggested that the use of a validated *or* non-validated FFQ did not significantly alter the risk associations. Furthermore, dietary changes after baseline collection can have an impact on the associations and repeated assessments of diet were not carried out in any of the cohort studies. However, measurement errors are generally non-differential in observational studies, which would most likely attenuate the association.

Second, the inherent problems of residual confounders stemming from observational studies are of concern, because of the inability to fully adjust for various confounders. Persons eating higher levels of VFs may be more likely to take up other healthy lifestyles, such as a lower prevalence of obesity and alcohol use, less physical inactivity and tobacco smoking [45], and a less prevalence of hypertension [46]. However, most included studies have adjusted for a wide range of potential confounders. For example, 16 studies adjusted for tobacco smoking, 15 for BMI and 4 for hypertension, however, no studies adjusted for physical activity. Stratified analyses indicated that risk associations were significantly changed by adjustment for BMI, but not by adjustment for smoking, alcohol use, total energy intake and history of hypertension. Recently, meta-analyses have indicated that increased VF consumption decreases the risk for obesity [47], and increased BMI is associated with increased risk of RCC both for men and women [5]. These results indicated that BMI may have a residual confounding effect on the association between consumption of VFs and RCC risk.

Third, there was significant heterogeneity among studies. We assessed intake of total VF intake due to the relatively large number of studies on this topic. However, total VF intake includes cruciferous vegetables (such as, broccoli, Brussels sprouts, cabbage), which are rich sources of glucosinolates. Cruciferous vegetables may inhibit carcinogen-activating enzymes, detoxify carcinogens, and thus lower the occurrence of cancer [48]. In addition, some foods are culinary vegetables but are classified botanically as fruits, such as cucumbers, peppers, squash, and tomatoes. Various studies from different regions, ethnicities and time periods might use different VFs in their classifications and types. To cover this aspect, we included studies assessing “all” or “total” fruits or vegetables, thus providing one explanation for the heterogeneity across studies. In addition, higher heterogeneity was observed in dose-risk analyses, which may be due to unit conversions. Our subgroup analyses showed that cohort studies had little variability, whereas significant heterogeneity was observed among case-control studies. Furthermore, results from meta-regression analyses found that adjustment for BMI might partially (33.5% for vegetables and 60.1% for fruits) account for the observed heterogeneity. Additionally, we should be cautious of results from the non-linear association between consumption of vegetables and RCC risk because of the significant heterogeneity and low number of studies included, especially in the high doses of consumption categories (> 4 servings/day).

Finally, we did not consider the gray articles (small studies with null results) since they tend to be unpublished. Although neither Egger's nor Begg's test provided evidence of such bias, certain of publication bias may exist. This bias might exaggerate the protective effects of VF intake on RCC risk, these effects thus should be treated cautiously.

Collectively, our analysis indicated that a high intake of VF may lower the risk of RCC development. Because of the measurement errors of exposure assessment, the high heterogeneity across studies, and unmeasured confounding factors, further investigation with good designs are needed.

## MATERIALS AND METHODS

This meta-analysis evaluates the association between consumption of VF with RCC following the criteria set out by the Preferred reporting items for systematic reviews and meta-analysis guidelines [33]. There are no ethical issues involved in our study because our data were from published studies.

### Data sources and study identification

Two investigators (J.Z.K. and Z.S.J.) independently screened the original articles published in English language in the two databases of MEDLINE (from January 1, 1966), EMBASE (from January 1, 1974) and Web of science (from January 1, 1950) up to August 31, 2016. We used the following MeSH terms and Text Words: 1) “kidney” OR “renal”; 2) “carcinoma” “cancer” or “neoplasm” OR “neoplasia”; 3) “nutrition” OR “diet” OR “lifestyle” OR “intake” OR “ consumption” OR “fruit” OR “vegetable”; and 4) “case-control” OR “cohort” OR “retrospective” OR “prospective” OR “longitudinal”. Moreover, the reference lists of the included articles and published reviews were also screened and hand-searched. We did not consider abstracts or unpublished reports.

### Study selection

For this meta-analysis, we used the studies which evaluate fruit or vegetable groups classified as “all” or “total”. We did not include exposures presented as raw vegetables, green-yellow vegetables, cooked vegetables, green leaf vegetables, other vegetables, citrus fruit, or other specific types of fruits. But we included studies which reported “fresh vegetables” or “fresh fruit”, because fresh vegetables or fruit accounts for a very high proportion of the total consumption [50]. Two authors (J.Z.K. and Z.S.J.) independently reviewed all the retrieved studies to determine if they meet the inclusion criteria. Disagreements were settled through consensus with a third investigator (Y.J.J.).

The study inclusion criteria were:

- being a case-control or cohort design;
- presenting data for the association between total vegetables and/or fruits and RCC risk;
- reporting results in terms of adjusted estimates (at least for age) for the relative risk (RR) [e.g. hazard ratio, risk ratio or odds ratio (OR)] and 95% confidence interval (CI).

Non peer-reviewed articles, animal and mechanistic studies, ecologic assessments and correlation studies were not included for analysis. In case more than one articles on the association between intake of fruits and vegetables and RCC were identified, the most recent report was selected for our analysis.

### Data extraction

From each study, the following information was independently extracted by two researchers (J.Z.K. and Z.S.J.): first author's last name, study design, publication year, follow-up duration in cohort study, geographic locations where the study was carried out, number of cases, size of cohorts/number of controls, definition of controls, dietary data ascertainment (types and whether it was validated), exposure contrast, the RR estimates with their 95% CI for the highest vs. the lowest level and adjustment variables. When different types of adjusted RRs were presented, we extracted the one that controlled for the most confounders. Differences in data extraction between investigators were unusual and were resolved by consensus.

### Quality assessment

For each publication, the quality score was assessed by using the Newcastle-Ottawa quality assessment Scale (NOS) [51], which assigned a score of total 9 points (9 representing the highest quality) for individual study following this criteria: 4 items for selection, 2 items for comparability, and 3 items for exposure ( case-control study)/outcome (cohort study) assessment. We decided to assign two stars in the comparability section only when a study is adjusted for at least two of three main risk factors: tobacco smoking, hypertension and BMI. A total score ≥ 7 indicates high quality study. To avoid selection bias, no study was rejected because of these quality criteria.

### Statistical methods

We pooled RR estimates and 95% CIs for the comparison between the study-specific highest category of consumption versus the lowest, linear and non-linear dose-responses using the DerSimonian and Laird random-effects model, which incorporates both within- and between-study variability [52].

Heterogeneity was assessed using the Cochran Q (results were defined heterogeneous for *p* < 0.10) and I^2^ statistics (results explain the amount of total variation among studies). For the I^2^ statistic, heterogeneity was interpreted as absent (0%–25%), low (25.1%–50%), moderate (50.1%–75%), or high (75.1%–100%) [53]. Subgroup analyses and meta-regression analyses were carried out according to study design, geographic location, type of food frequency questionnaire (FFQ), exposure data available, study quality score, and confounders (adjustments for smoking, alcohol use, body mass index [BMI], history of hypertension and dietary energy intake). Sensitivity analysis that investigates the influences of each individual study on the summary results was performed by omitting one study at a time.

To conduct dose-response meta-analyses, published results were transformed into a common scale, which are expressed as increment of 1 serving/day of consumption. For one study which presented the intake per given unit of energy intake, we rescaled it using the mean energy intake provided [12]. For studies that reported intakes as grams, we converted an 80 g as 1 serving size according to other meta-analyses of fruit and vegetable intake and cancer risk. [50]. When results for intakes were reported as a continuous variable (e.g., for 40 g/d increase in intake), we rescaled the RR to a 1 serving per day increase in intakes [19], and included them only in the dose-response analysis. We used the methods of generalized least-squares trend estimation (GLST) analysis [54, 55], which requires that at least three categories of intake and the number of cases and person-years or non-cases per category is known. Whenever reported, the mean or median intake by category was assigned to the corresponding RR. We assigned the median in each category by calculating the average of the lower and upper bound. When the lowest and highest category was open-ended, we assumed the open-ended interval length was equal to the adjacent interval.

To examine the non-linear dose-response relationship, we carried out the best-fitting second-order fractional polynomial models [56]. The model with the lowest deviance was selected, and using a likelihood ratio test to evaluate the difference between the nonlinear and linear models [56].

Publication bias was assessed by using funnel plots and the further Begg's adjusted rank correlation and Egger's regression asymmetry test [57, 58]. All statistical analyses were performed using STATA (College Station, TX, USA; version 11.0) and R-package (Version 2.11.0 beta, R Development Core Team, NJ) statistical softwares. A two-tailed *P* value of < 0.05 represents significance.

## SUPPLEMENTARY MATERIALS FIGURES AND TABLES


