# Correction: Consumption of fruits and vegetables and risk of renal cell carcinoma: a meta-analysis of observational studies

**DOI:** 10.18632/oncotarget.25969

**Published:** 2018-08-07

**Authors:** Shaojin Zhang, Zhankui Jia, Zechen Yan, Jinjian Yang

**Affiliations:** ^1^ Department of Urology Surgery, The First Affiliated Hospital, Zhengzhou University, Zhengzhou, Henan Province, China

**This article has been corrected:** The correct author name is given below:

**Shaojin Zhang**

Original article: Oncotarget. 2017; 8:27892-27903. https://doi.org/10.18632/oncotarget.15841

