# Supplementary material for: Consumption of fruits and vegetables and risk of renal cell carcinoma: a meta-analysis of observational studies
Source: Oncotarget. 2017 Mar 2;8(17):27892–903. doi: 10.18632/oncotarget.15841 (PMC5438616; doi:10.18632/oncotarget.15841)
Supplement: Supplementary file 1 [file oncotarget-08-27892-s001.pdf]

Consumption of fruits and vegetables and risk of renal cell carcinoma: a meta-analysis of observational studies

Supplementary Materials

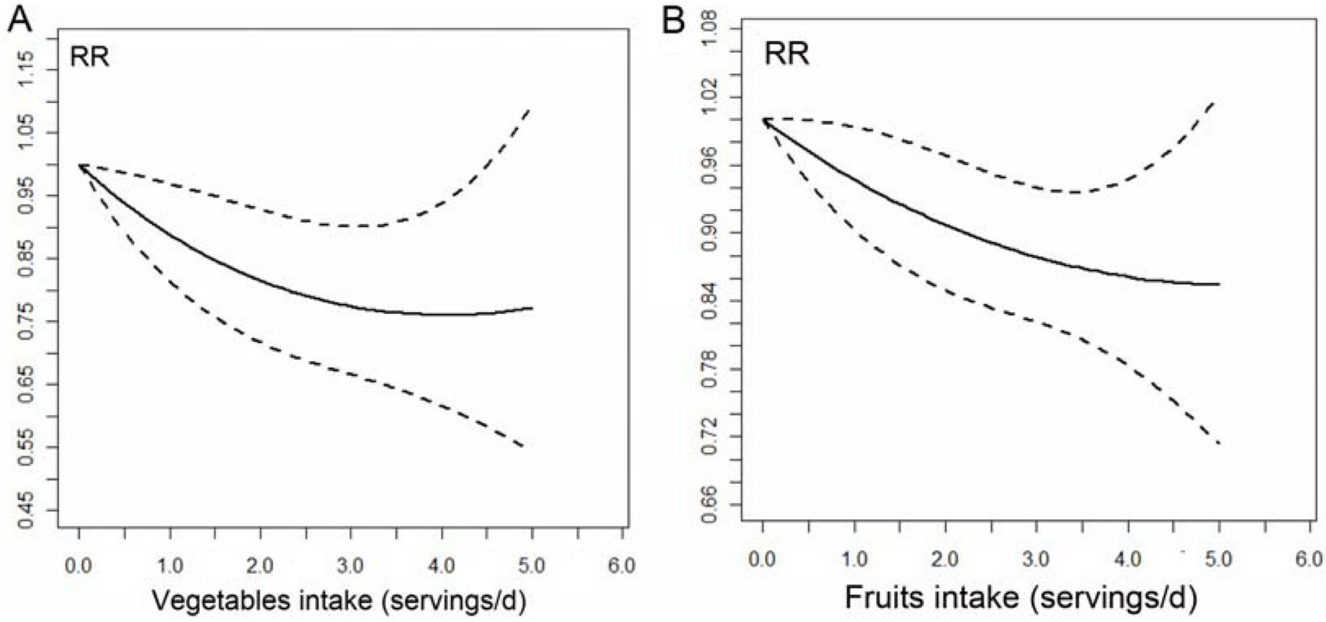

Supplementary Figure 1: Relative risk and the corresponding 95% confidence interval describing the non-linear association of intake of vegetables (**A**) and fruit (**B**) and the risk of renal cell carcinoma. The *P* values for the best-fitting second-order fractional polynomial regression model were 0.001 for vegetables and 0.221 for fruits intake, respectively.

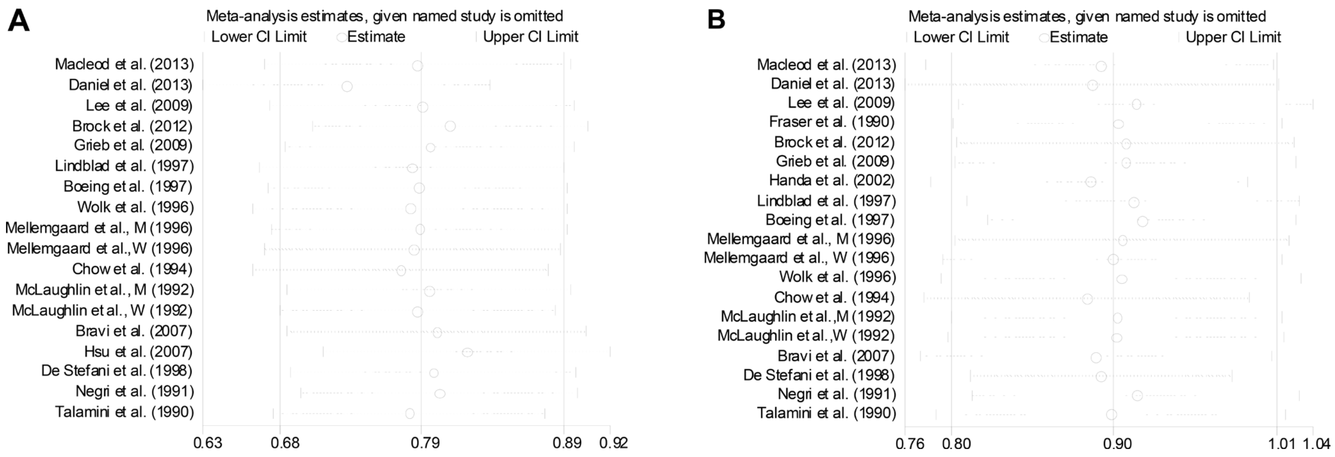

Supplementary Figure 2: Sensitivity analyses for the association between intake of vegetables (**A**) and fruit (**B**) and the risk of renal cell carcinoma.

Supplementary Table 1: Quality assessment according to the Newcastle-Ottawa scale

Author	Selection	Comparability	Outcome	Exposure	Score
**Case-control**					
Brock et al., 2012	4	2	/	3	9
Grieb et al., 2009	4	2	/	2	8
Handa & Kreiger, 2002	3	2	/	2	7
Lindblad et al., 1997	3	2	/	2	7
Boeing et al., 1997	3	1	/	2	6
Wolk et al., 1996	3	2	/	2	7
Mellemgaard et al., 1996	4	2	/	3	9
Chow et al., 1994	3	2	/	3	8
McLaughlin et al., 1992	4	2	/	3	9
Bravi et al., 2007	2	2	/	3	7
Hsu et al., 2007	2	2	/	3	7
De Stefani et al., 1998	3	1	/	3	7
Negri et al., 1991	3	1	/	3	7
Talamini et al., 1990	3	1	/	1	5
**Cohort**					
Macleod et al., 2013,	4	2	3	/	9
Daniel et al., 2013,	4	2	3	/	9
Lee et al., 2009	4	2	3	/	9
Weikert et al., 2006	4	2	3	/	9
Fraser et al., 1990	2	1	3	/	6
